# Supplementary material for: Alpha-Gal Bound Aptamer and Vancomycin Synergistically Reduce Staphylococcus aureus Infection In Vivo
Source: Microorganisms. 2023 Jul 8;11(7):1776. doi: 10.3390/microorganisms11071776 (PMC10383818; doi:10.3390/microorganisms11071776)
Supplement: Supplementary file 1 [file microorganisms-11-01776-s001.zip › microorganisms-2483219-supplementary.pdf]

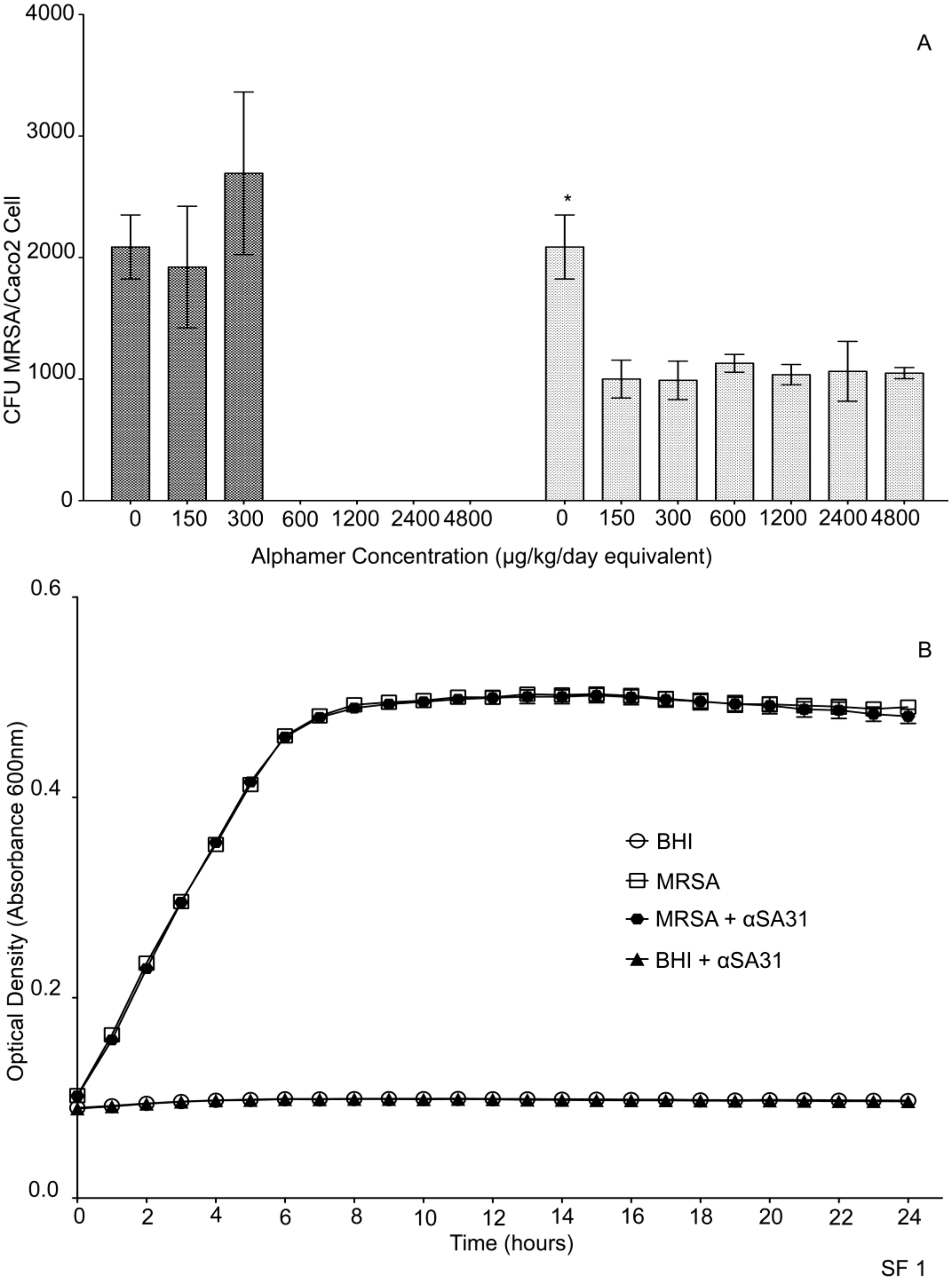

Supplemental Figure S1. αSA31 does not affect MRSA growth. A) MRSA was incubated in the presence or absence of drug and adhesion to Caco2 cells was measured using and anti-flu aptamer (dark grey) or α SA31 (light grey). B) MRSA growth was recorded in the presence or absence of 12.5 ng/μL αSA31 in BHI media + 20% GTKO mouse serum.

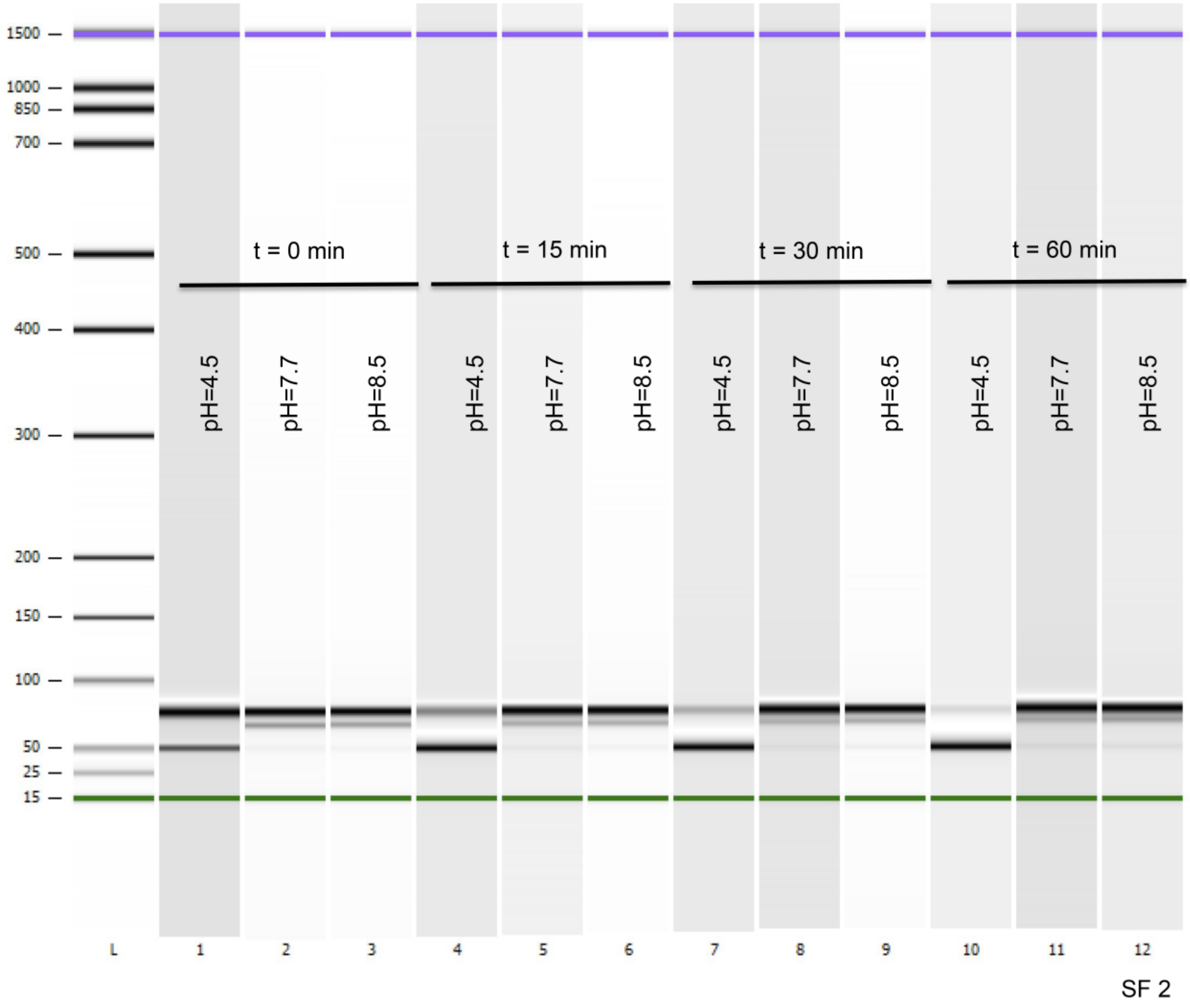

Supplemental Figure S2. Alphamer stability across pH. αSA31 was incubated in acidic, neutral, and basic conditions for 1 hr and then analyzed using a bioanalyzer. Electropherograms were converted to gel images. In neutral to basic conditions the majority of aptamer is bound to α-gal.

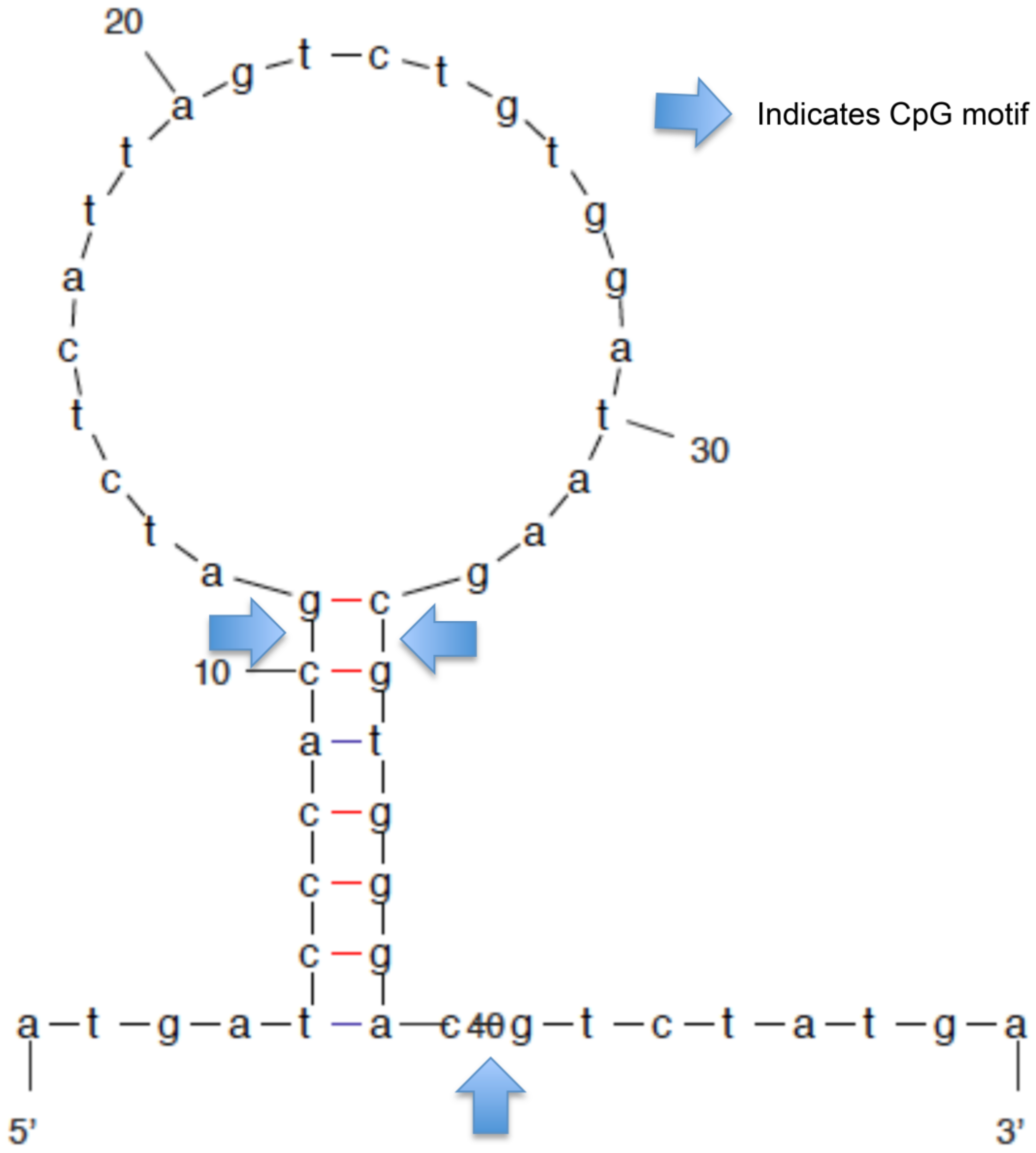

SF 3

Supplemental Figure S3. 2D structural analysis of SA31 aptamer. Folding analysis of SA31 performed using Mfold web server for nucleic acid folding and hybridization prediction [43].

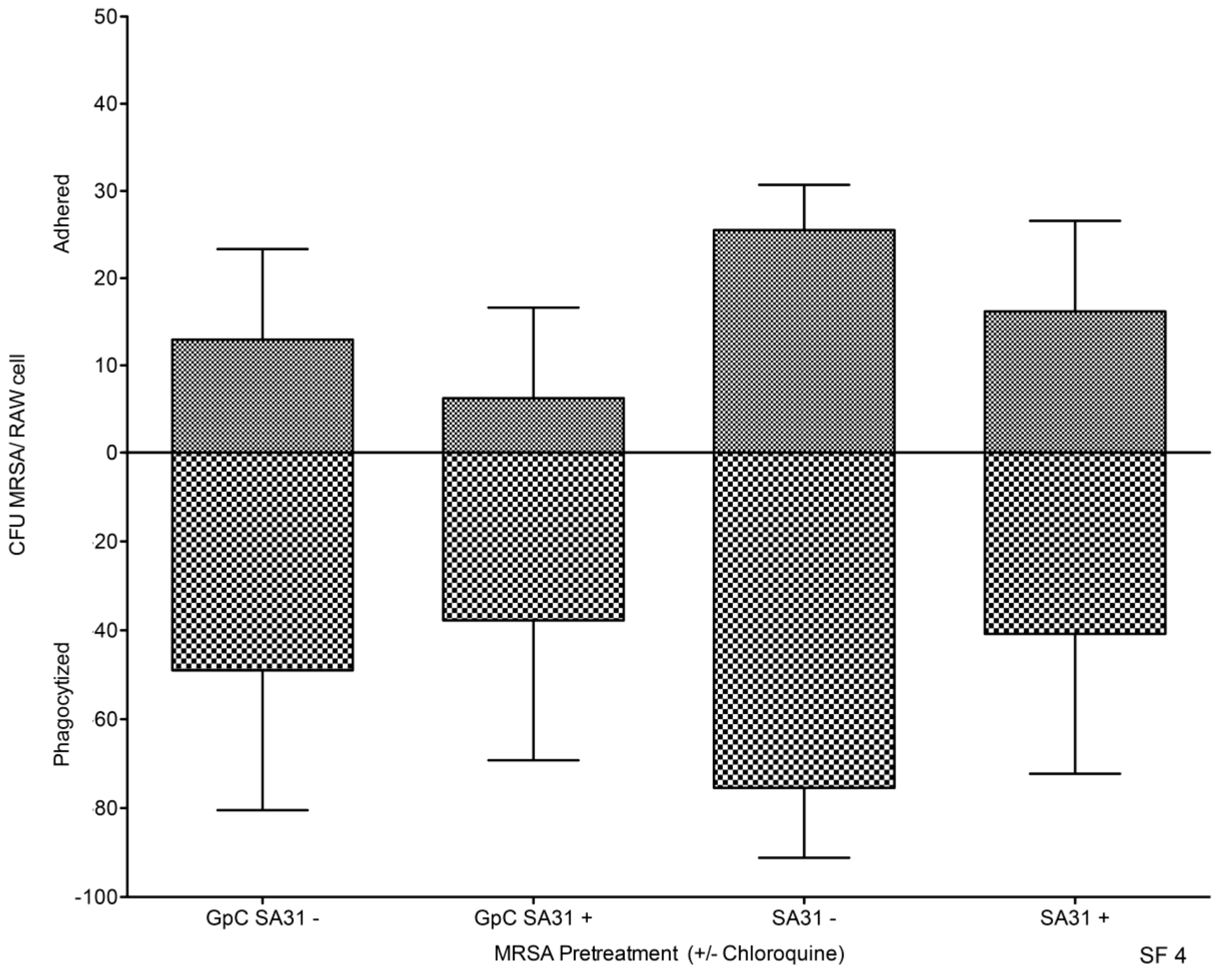

Supplemental Figure S4. CpGs not involved in increased phagocytosis. MRSA was incubated in the presence or absence of 20% GTKO mouse serum in PBS and then incubated with/without 150 ng/ml aptamer prior to addition to RAW cells at MOI = 100 in the presence/absence of 10 μg/ml chloroquine. Adhered and phagocytized CFU MRSA per cell was measured using qPCR. No significant difference was observed between treatments.

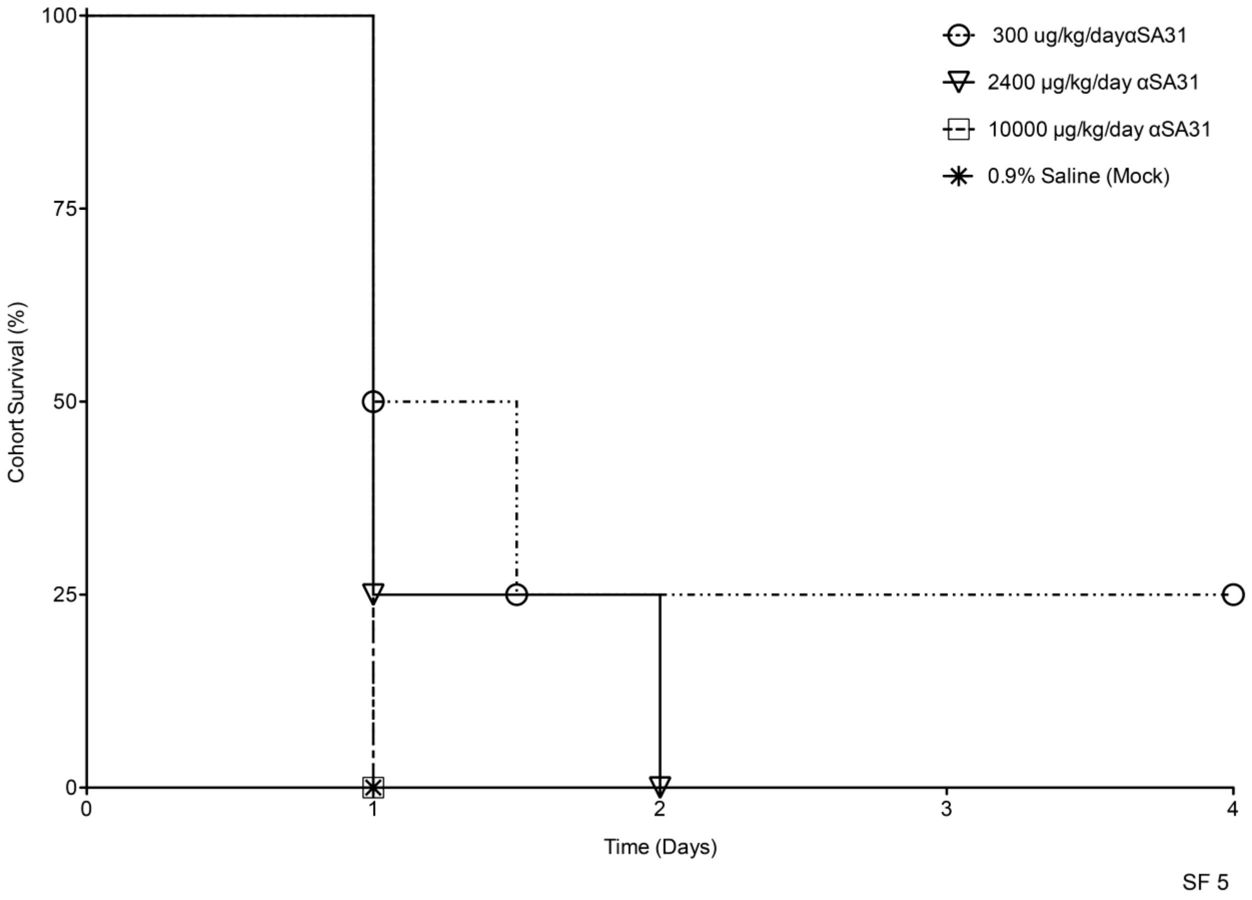

Supplemental Figure S5. Survival of Mice Infected with MRSA and Treated with αSA31. Mice (n=4) were infected via tail vein IV with 10^9 CFU MRSA and then treated with αSA31 twice daily IV. [a] The surviving mouse in the 300 μg/kg/day showed evidence of tail infection instead of sepsis.

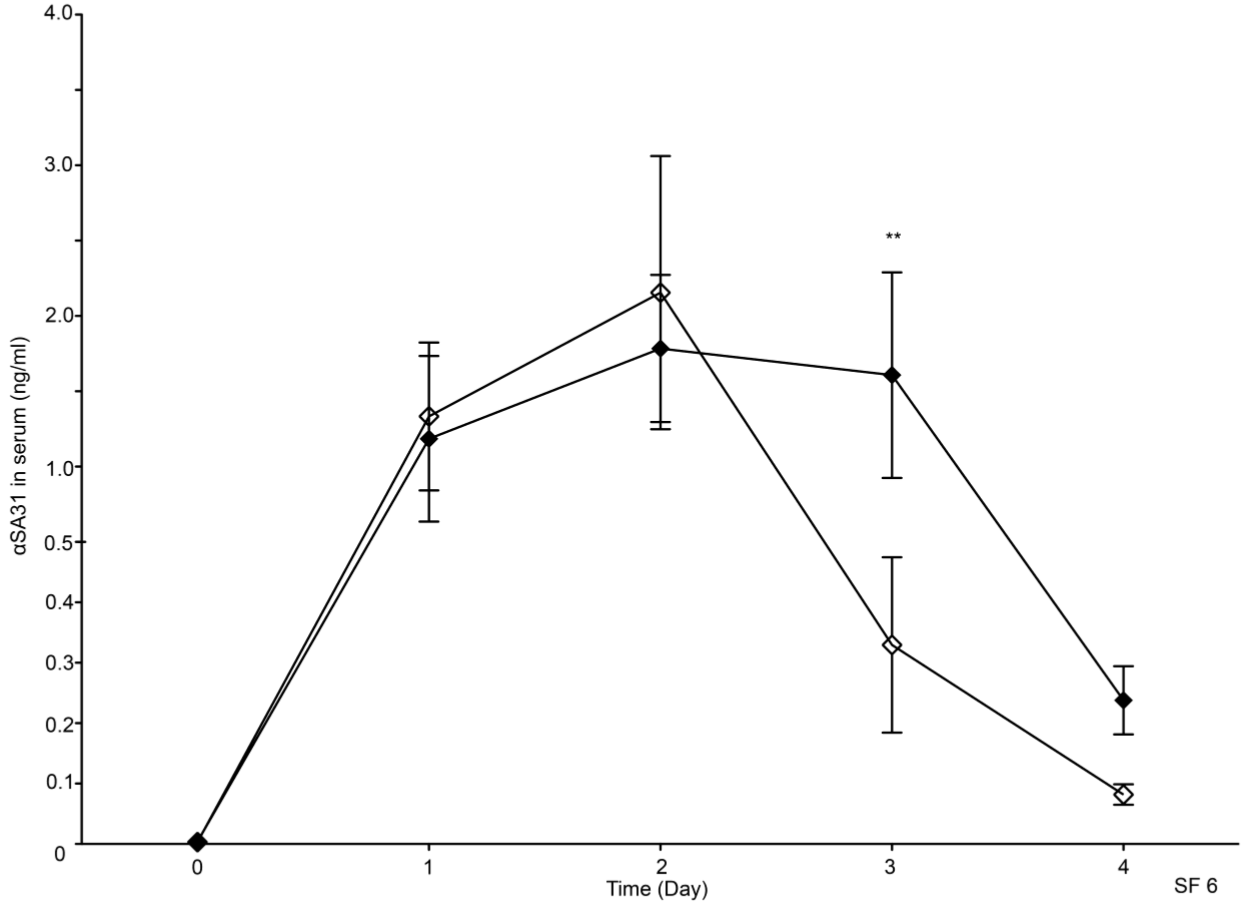

Supplemental Figure S6. Serum Concentration of αSA31 in uninfected and MRSA infected mice. Uninfected mice (black diamond) (n=4) were injected twice daily at 10,000 μg/kg/day αSA31 and infected mice (open diamond) were injected with $1x10^9$ CFU of MRSA and treated with αSA31 plus 60 mg/kg/day Vancomycin. Serum was collected prior to morning dosage. The serum concentration of αSA31 was determined by qPCR. ** Indicates a significant difference ($P<0.01$) between infected vs. uninfected mice on day 3. Statistics are from a 2-way Repeated Measures ANOVA using Prism 5 for Mac.

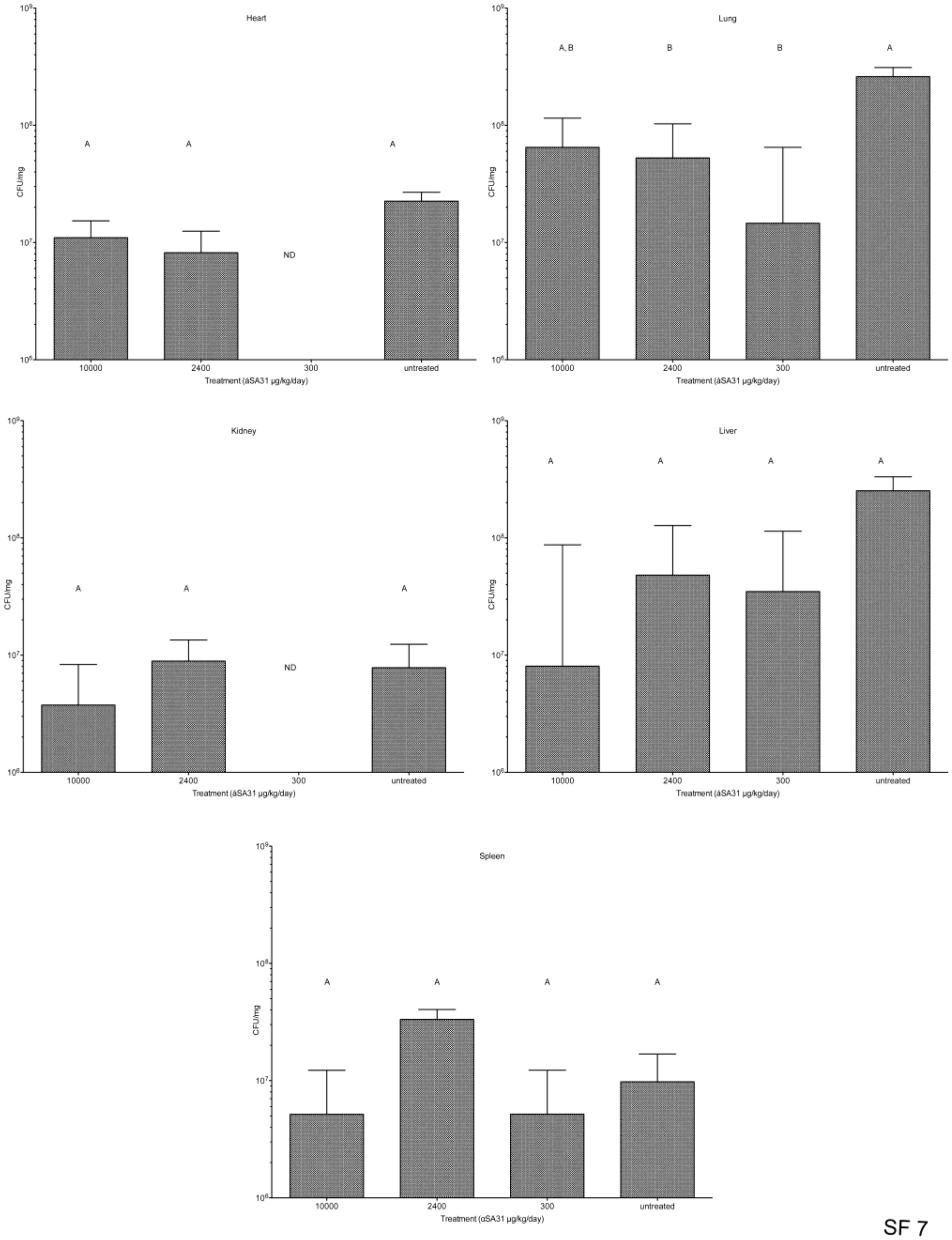

SF 7

Supplemental Figure S7. Organ data from mice treated with SA31 alone.
The spleen, lung, liver, heart, and kidney were collected at necropsy. Half of the organs were homogenized, and lysed to determine the bacterial load in CFU/mg organ by qPCR. Significance was established using nonparametric analysis followed by Tukey’s HSD post-test in JMP10 for CFU/mg organ (n=4). Bars not connected by the same letter are significantly different.

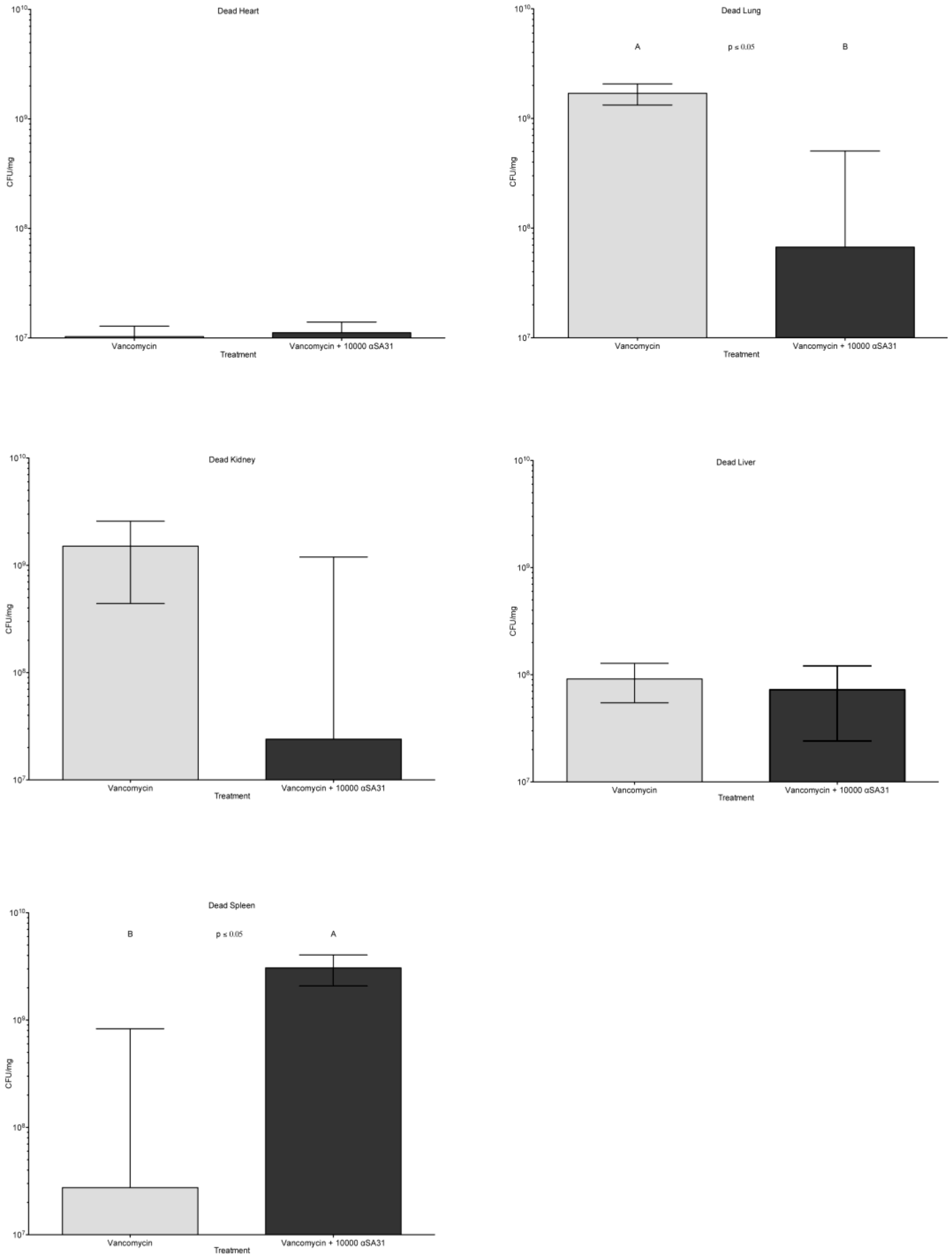

SF 8

Supplemental Figure S8. Organ data from mice treated with vancomycin or vancomycin plus αSA31 that died. The spleen, lung, liver, heart, and kidney were collected at necropsy. Half of the organs were homogenized and lysed to determine the bacterial load in CFU/mg organ by qPCR. Significance was established using student's t-test in JMP10 for CFU/mg organ with results segregated based on mouse survival (n=12). Bars not connected by the same letter are significantly different.

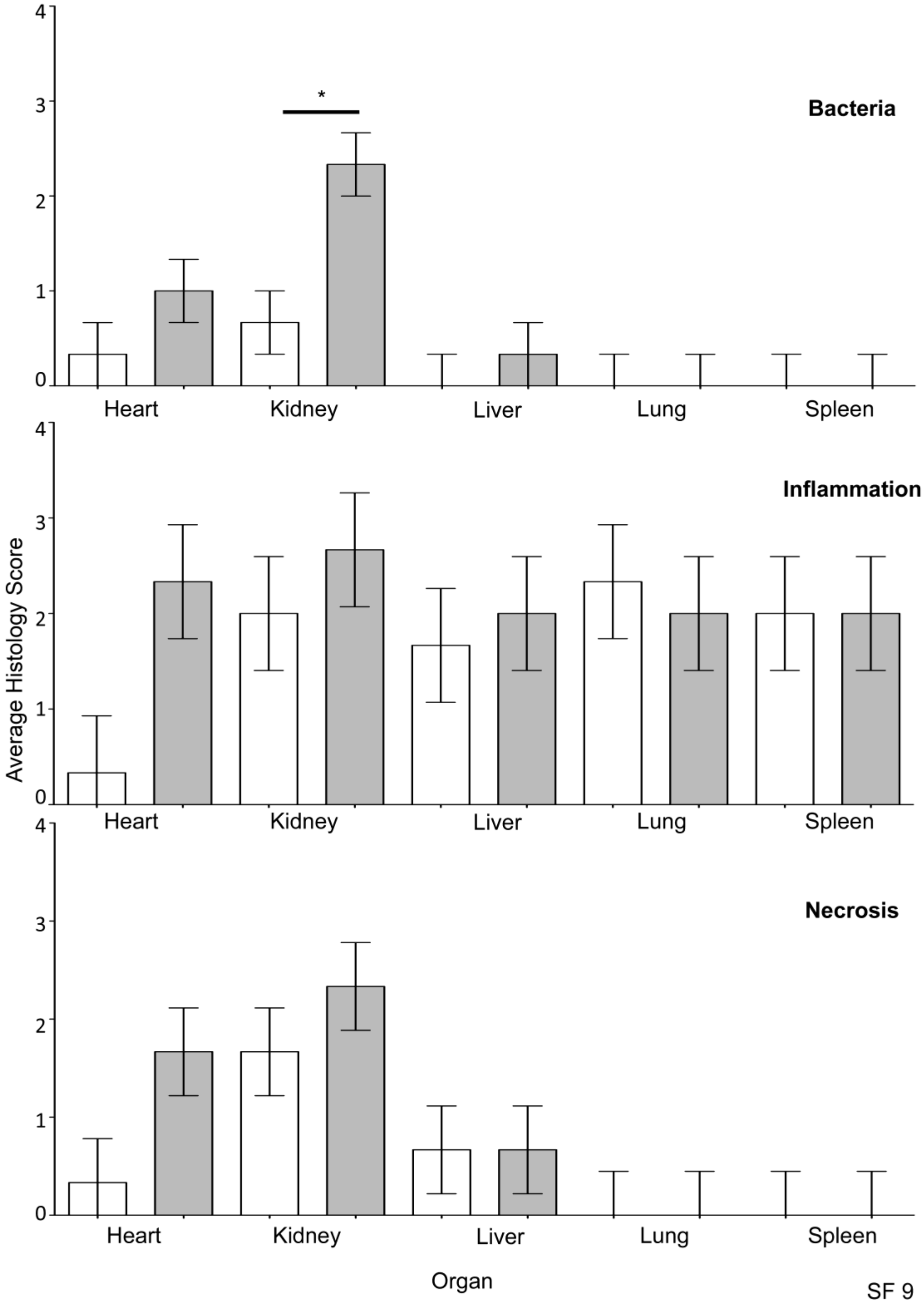

Supplemental Figure S9. Histology Scores. Histology was performed and scored by a single pathologist (L. Woods) blinded to the treatment groups. The scores were averaged for bacteria, inflammation, and necrosis (B/I/N) on a scale of 0-4 with 0 being healthy tissue with no bacteria and 4 being severely damaged tissue or numerous bacteria. Statics were performed in JMP10 for mac using a 2-way ANOVA and Tukey's HSD post-test (n=3).
